# Circ_0001721 enhances doxorubicin resistance and promotes tumorigenesis in osteosarcoma through miR-758/TCF4 axis

**DOI:** 10.1186/s12935-021-02016-5

**Published:** 2021-07-02

**Authors:** Huapeng Guan, Hao Xu, Jinshui Chen, Weishan Wu, Dongfeng Chen, Yungang Chen, Jianzhong Sun

**Affiliations:** 1 Department of Orthopedics, 900Th Hospital of the Joint Logistics Team (Fuzhou General Hospital Affiliated to Fujian Medical University), Fuzhou, 350025 Fujian China; 2 Department of Orthopedics, Affiliated Hospital of Shandong University of Traditional Chinese Medicine, Jinan, 250000 Shandong China; 3 Department of Orthopedics, Shanghai Baoshan Hospital of Integrated Traditional Chinese and Western Medicine, No.181 Youyi Road, Baoshan District, Shanghai, 201999 P.R. China

**Keywords:** Osteosarcoma, circ_0001721, miR-758, TCF4, Doxorubicin, Tumorigenesis

## Abstract

**Background:**

Osteosarcoma (OS) is a common type of bone malignancy that often occurs in children and adolescents. Chemoresistance is a huge barrier to cancer therapy. This study aimed to investigate the role and potential mechanism of circ_0001721 in doxorubicin (DXR) resistance and OS development.

**Methods:**

The levels of circ_0001721, miR-758 and transcription factor 4 (TCF4) were detected by quantitative real-time polymerase chain reaction or western blot assay. Cell Counting Kit-8 (CCK-8) assay was used to calculate the half inhibition concentration (IC_50_) of DXR and assess cell viability. Cell migration and invasion were evaluated by transwell assay. Cell apoptosis was monitored by flow cytometry. The levels of multidrug resistance-related and Wnt/β-catenin pathway-related proteins were measured by western blot assay. The interaction among circ_0001721, miR-758 and TCF4 were confirmed by dual-luciferase reporter assay, RNA immunoprecipitation assay or RNA pull-down assay. The xenograft model was established to analyze tumor growth in vivo.

**Results:**

Circ_0001721 and TCF4 were upregulated, whereas miR-758 was down-regulated in DXR-resistant OS tissues and cells. Circ_0001721 silence reduced DXR resistance of KHOS/DXR and MG63/DXR cells. Circ_0001721 regulated DXR resistance via sponging miR-758. Moreover, miR-758 modulated DXR resistance by targeting TCF4. Besides, circ_0001721 knockdown inhibited tumor growth in vivo.

**Conclusion:**

Circ_0001721 potentiated DXR resistance and facilitated the progression of OS by regulating miR-758/TCF4 axis, which provides promising therapeutic targets for OS treatment.

## Introduction

Osteosarcoma (OS) is common malignant bone cancer in children and adolescents [1]. With the improvement of OS treatment strategies, the therapy of OS is not only dependent on surgical resection, but the introduction of chemotherapy has improved the prognosis of OS patients [2]. At present, the 5-year survival rate of patients with non-metastatic OS has reached about 70% [3]. Nevertheless, the emergence of chemoresistance has become a significant obstacle to the treatment of osteosarcoma [4]. Tumor microenvironment of osteosarcoma plays an important role in the occurrence and development of the disease [5, 6]. In addition, in vivo models allow us to further understand the crosstalk between tumors and other tissues [7]. Thus, elucidating the mechanism of chemoresistance and discovering specific biomarkers are essential to improve the survival rate of osteosarcoma.

Circular RNAs (circRNAs) are a new type of non-coding RNAs with a covalent closed-loop structure that makes them very stable [8]. Accumulating evidence has testified that circRNAs exert a critical effect on the development and progression of various cancers [9]. Previous researches manifested that circRNAs are abnormally regulated in OS, and the circRNA imbalance is related to the pathogenesis of OS [10]. For example, circ_0000502 was abnormally up-regulated in OS, and its overexpression facilitated OS progression by regulating microRNA-1238 [11]. CircSAMD4A was upregulated and accelerated tumor progression in OS via regulating the microRNA-1244/MDM2 pathway [12]. In addition, circ_0001721 is a transcript of cyclin dependent kinase 14 (CDK14), and the upregulation of circ_0001721 is closely related to the poor prognosis of OS [13]. Nevertheless, the role of circ_0001721 in chemoresistance of OS remains unknown.

MicroRNAs (miRNAs) are a class of endogenous non-coding RNAs with a length of 19–25 nucleotides and play a vital role in the development and progression of tumors [14]. Increasing evidence has revealed that circRNA can regulate biological processes by serving as miRNA sponge [15]. For example, circ_0009910 exerted a carcinogenesis effect on OS via sponging miR-449a to upregulate IL6R [16]. Wu et al. presented that circTADA2A sponged miR-203a-3p to facilitate OS progression through regulation of CREB3 [17]. Therefore, the molecular mechanism of circ_0001721 needs further investigation.

Transcription factor 4 (TCF4) classified in the T-cell factor/lymphoid enhancer factor family is an essential downstream effector of the Wnt pathway [18]. Previous studies have demonstrated that TCF4 alternative splicing shows underlying tumor-promoting properties in various malignancies [19].

In this research, we further verified that circ_0001721 was remarkably up-regulated in OS tissues and cells. In addition, the function and potential mechanism of circ_0001721 in chemoresistance and tumor progression of OS were further explored.

## Materials and methods

### Specimen collection

Fifty-one OS tissues and fifty-one adjacent normal bone tissues within 0.5 cm around the tumors were obtained from OS patients who did not receive any preoperative treatment at 900th Hospital of the Joint Logistics Team. Meanwhile, fifty-one OS patients who were sensitive to doxorubicin treatment (Chemosensitive) and fifty-one OS patients who were resistant to doxorubicin treatment (Chemoresistant) after receiving doxorubicin chemotherapy were recruited from 900th Hospital of the Joint Logistics Team. The resistance of OS patients to chemotherapy was classified according to the Huvos scoring system. This research was ratified by the Ethics Committee of 900th Hospital of the Joint Logistics Team. All participants signed written informed consent. The characteristics of patients and patients with chemosensitive, chemoresistant in osteosarcoma are presented in Table 1.

**Table 1:** Characteristics of patients and patients with chemosensitive, chemoresistant in osteosarcoma

Characteristics	Patients (n = 51)	Chemosensitive (n = 51)	Chemoresistant (n = 51)
Gender			
Female	25	26	23
Male	26	25	28
Age (years)			
< 50	28	25	24
≥ 50	23	26	27
Histological type			
Well, moderate	26	27	25
Poor	25	24	26
TNM stage			
I–II	16	20	21
III–IV	35	31	30
Tumor size			
≤ 5 cm	32	33	30
> 5 cm	19	18	21
Lymph node metastasis			
N0–N1	22	20	23
N2–N3	29	31	28
Distant metastasis			
Absence	31	33	30
Present	20	18	21

### Cell culture and treatment

Human fetal osteoblastic cells hFOB1.19 and OS cell lines (KHOS and MG63) were bought from American Type Culture Collection (ATCC, Manassas, VA, USA). KHOS and MG63 cells were exposed to increasing doses of doxorubicin (DXR) (Solarbio, Beijing, China) to generate DXR-resistant cell lines (KHOS/DXR and MG63/DXR). All OS cells were incubated in Dulbecco’s Modified Eagle Medium (DMEM; Gibco, Carlsbad, CA, USA) supplemented with 10% fetal bovine serum (FBS; Gibco) at 37 °C with 5% CO_2_. Additionally, hFOB1.19 cells were cultured in RPMI-1640 (Gibco) containing 10% FBS (Gibco) at 33.5 °C with 5% CO_2_.

### Cell transfection

Small interfering RNA (siRNA) against circ_0001721 (si-circ#1, si-circ#2 and si-circ#3), the siRNA negative control (si-NC), miR-758 mimics (miR-758), the mimic control (miR-NC), circ_0001721 overexpression vector (circ_0001721), TCF4 overexpression vector (TCF4), the empty overexpression vector (pcDNA), miR-758 inhibitor (in-miR-758) and the control (in-miR-NC) were commercially obtained from Ribobio (Guangzhou, China). The vectors and oligonucleotides were transfected into KHOS/DXR and MG63/DXR cells using Lipofectamine 3000 (Invitrogen, Carlsbad, CA, USA).

### Quantitative real-time polymerase chain reaction (qRT-PCR)

After extracting RNA with Trizol (Invitrogen), complementary DNA (cDNA) was synthesized using HiScript II One Step RT-PCR Kit (Vazyme, Nanjing, China) or miRNA 1st Strand cDNA Synthesis Kit (Vazyme). Then, AceQ qPCR SYBR Green Master Mix (Vazyme) was used for quantitative PCR. β-actin and U6 were taken as internal controls. Primers were shown below: circ_0001721-F: 5’-CACCTAAAGTTAGGCGGCAC-3’, circ_0001721-R: 5’-TGGGTCAAAAGTGCTCTGTG-3’; miR-758-F: 5’-ACACTCCAGCTGGGTTTGTGACCTGGTCCA-3’, miR-758-R: 5’-TGGTGTCGTGGAGTCG-3’; TCF4-F: 5’-CAAGCACTGCCGACTACAATA-3’, TCF4-R: 5’-CCAGGCTGATTCATCCCACTG-3’; β-actin-F: 5’- TGGATCAGCAAGCAGGAGTA -3’, β-actin-R: 5’- TCGGCCACATTGTGAACTTT -3’; U6-F: 5’-CTCGCTTCGGCAGCACA-3’, U6-R: 5’-AACGCTTCACGAATTTGCGT-3’.

### Cell viability assay

Cells (3 × 10^3^) were seeded into 96-well plates and stimulated with escalating doses of DXR for 48 h. At the same time, transfected DXR-resistant cells (3 × 10^3^) were cultured in 96-well plates for 0 h, 24 h, 48 h or 72 h. Subsequently, cells were interacted with 10 μL Cell Counting Kit-8 (CCK-8) solution (Solarbio) for 2 h. Then, the optical density was measured at 450 nm using a Multi-Mode Reader (BioTek, Burlington, VT, USA). The half maximal inhibitory concentration (IC_50_) of DXR was the concentration of DXR when cell viability was reduced to 50%.

### Transwell assay

For cell migration assay, 1 × 10^5^ cells were placed in the upper chamber. After incubation for 24 h, the non-migrated cells were wiped off with a cotton swab, and the migrated cells were stained with 0.5% crystal violet. Then, the migrated cells were counted using a microscope. For cell invasion assay, transwell chambers were coated with Matrigel (BD Biosciences, San Diego, CA, USA), and the other procedures were the same as the migration assay.

### Flow cytometry

Transfected cells (1 × 10^6^) were plated in six-well plates and incubated with an AnnexinV-fluorescein isothiocyanate (AnnexinV-FITC)/Propidium Iodide (PI) Apoptosis Detection kit (Invitrogen). Next, the apoptosis rate was monitored using the CytoFLEX flow cytometer (Beckman Coulter, Miami, FL, USA).

### Western blot assay

Cells were lysed with RIPA buffer (Solarbio). After centrifugation and quantification, equal amounts of protein were separated by polyacrylamide gel electrophoresis and transferred to polyvinylidene fluoride (PVDF) membranes (Millipore, Billerica, MA, USA). Subsequently, the membranes were probed with primary antibodies against multidrug resistance associated protein 1 (MRP1) (ab233383, Abcam, Cambridge, UK), P-glycoprotein (P-gp) (ab129450, Abcam), lung resistance protein (LRP) (ab92544, Abcam), β-catenin (ab16051, Abcam), cyclin D1 (ab226977, Abcam), c-myc (ab39688, Abcam), TCF4 (ab185736, Abcam) and β-actin (ab8227, Abcam) at a dilution ratio of 1:1000. Next, the membranes interacted with secondary antibody (ab7090, Abcam). The signal intensity was tested by the enhanced chemiluminescence system (Millipore). Three independent experiments were performed on each sample.

### Dual-luciferase reporter assay

The sequences of circ_0001721 or TCF4 3’UTR containing the predicted miR-758 wild or mutant binding sites were inserted into pmirGLO vector (Promega, Madison, WI, USA) to generate circ_0001721 WT, circ_0001721 MUT, TCF4 3’UTR WT or TCF4 3’UTR MUT reporter. Then, the corresponding luciferase reporter and miR-758 or miR-NC were co-transfected into KHOS/DXR and MG63/DXR. Finally, the luciferase intensity was monitored using a Dual-Luciferase Reporter Assay Kit (Vazyme).

### RNA immunoprecipitation (RIP) assay

RIP analysis was performed with the EZ-Magna RIP kit (Millipore). First, KHOS/DXR and MG63/DXR cells transfected with miR-758 or miR-NC were lysed using RIP lysis buffer. Next, cell lysates were incubated with magnetic beads conjugated with Ago2 antibody or IgG antibody. Final, the enrichment of circ_0001721 was measured by qRT-PCR.

### RNA pull-down assay

Biotin-labeled circ_0001721 probe (Bio-circ_0001721 WT) and the control probe (Bio-circ_0001721 MUT) were purchased from RiboBio. Briefly, biotinylated probes were reacted with M-280 Streptavidin Dynabeads (Invitrogen) at 37 °C for 2 h to construct probe-coated beads. Next, the cells were lysed and incubated with probe-coated beads at 4 °C for 3 h. Finally, the level of miR-758 was examined by qRT-PCR.

### Xenograft assay

BALB/c nude mice (four-week-old) were randomly divided into two groups (n = 6 per group). MG63/DXR cells were introduced with the lentivirus carrying short hairpin RNA against circ_0001721 (sh-circ_0001721) or the negative control (sh-NC) constructed by RiboBio. Then, stably transfected cells (5 × 10^6^) were subcutaneously injected into the right-back of nude mice. Tumor volume was monitored every 7 days. After 28 days of inoculation, the mice were sacrificed, and tumor samples were weighted. The xenograft assay was approved by the Animal Research Committee of 900th Hospital of the Joint Logistics Team. Animal studies were performed in compliance with the ARRIVE guidelines and the Basel Declaration. All animals received humane care according to the National Institutes of Health (USA) guidelines.

### Statistical analysis

Data were expressed as mean ± standard deviation of three independent experiments by using Graphpad Prism 7.0 software (GraphPad, San Diego, CA, USA). Student’s* t*-test was used to analyze the differences between the two groups, and one-way analysis of variance was conducted to test the differences between multiple groups. Turkey’s post-hoc test was used to verify ANOVA for pairwise comparisons. *P* < 0.05 was regarded as statistically significant.

## Results

### Circ_0001721 was highly expressed in DXR-resistant OS tissues and cells

**Fig. 1:**
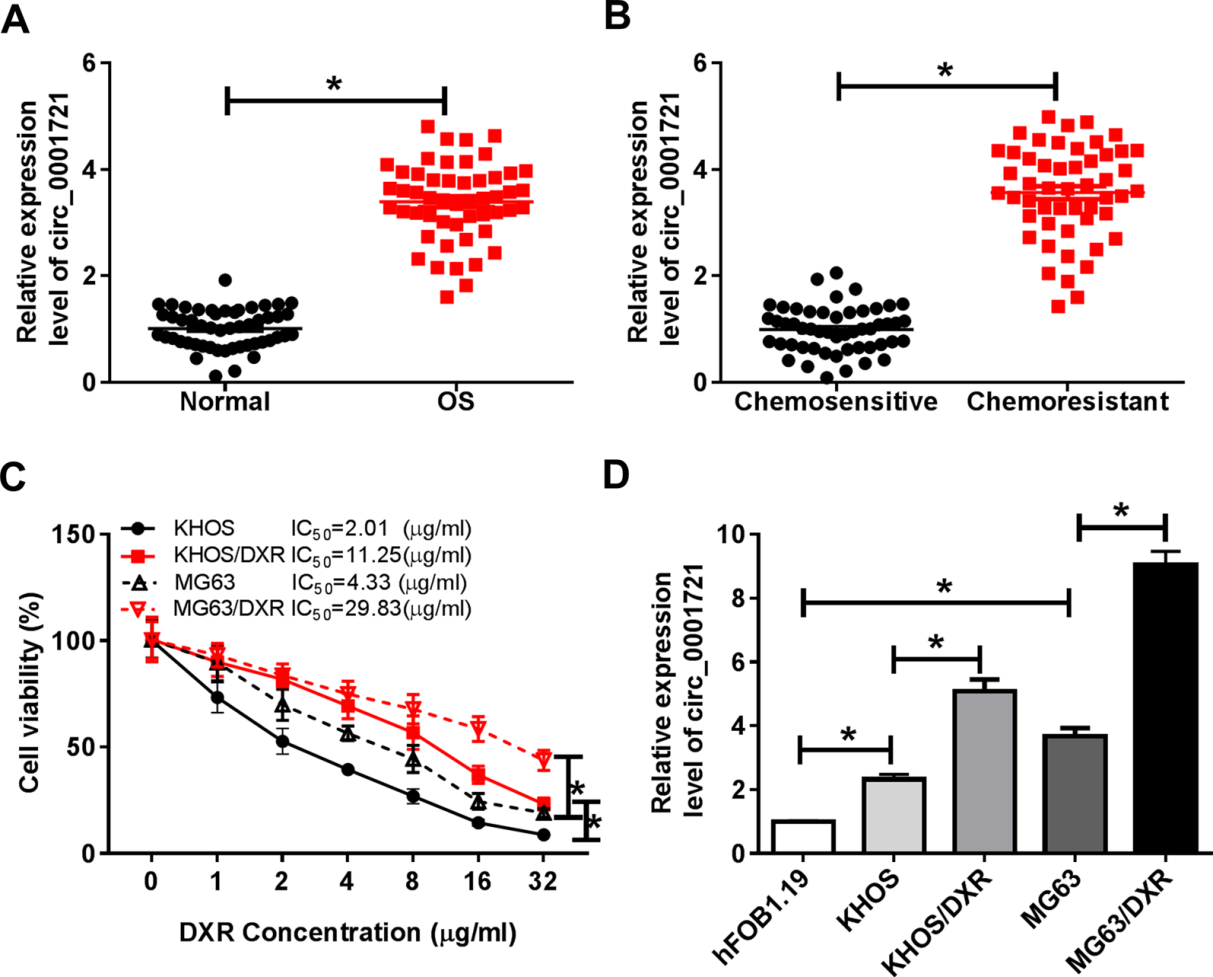
Circ_0001721 was highly expressed in DXR-resistant OS tissues and cells. **A** qRT-PCR was used to test the expression of circ_0001721 in OS tissues (n = 51) and adjacent normal tissues (n = 51). **B** Expression of circ_0001721 in Chemosensitive (n = 51) and Chemoresistant (n = 51) OS tissues was detected by qRT-PCR. **C** KHOS, KHOS/DXR, MG63 and MG63/DXR cells were treated with different concentrations of DXR, and IC_50_ values were determined by CCK-8 analysis. **D** Circ_0001721 expression was detected in hFOB1.19 cells, parental OS cells (KHOS and MG63) and DXR-resistant OS cells (KHOS/DXR and MG63/DXR) using qRT-PCR. *P < 0.05

To investigate the role of circ_0001721 in OS, we first examined the expression of circ_0001721 in OS tissues and adjacent normal tissues. The results indicated that circ_0001721 expression was distinctly increased in OS tissues relative to adjacent normal bone tissues (Fig. 1A). In addition, the expression of circ_0001721 was remarkably elevated in DXR-resistant OS patients compared with the Chemosensitive group (Fig. 1B). Moreover, the IC_50_ of DXR in KHOS/DXR and MG63/DXR cells was markedly higher than that of KHOS and MG63 cells, indicating that DXR-resistant cell lines were successfully constructed (Fig. 1C). Also, the expression of circ_0001721 in KHOS and MG63 cells was strikingly higher than that in hFOB1.19 cells, and prominently lower than that in DXR-resistant OS cells (Fig. 1D). These data suggested that circ_0001721 might play a promoting role in OS progression and drug resistance.

### Circ_0001721 knockdown impeded DXR resistance and tumor progression in DXR-resistant OS cells

**Fig. 2:**
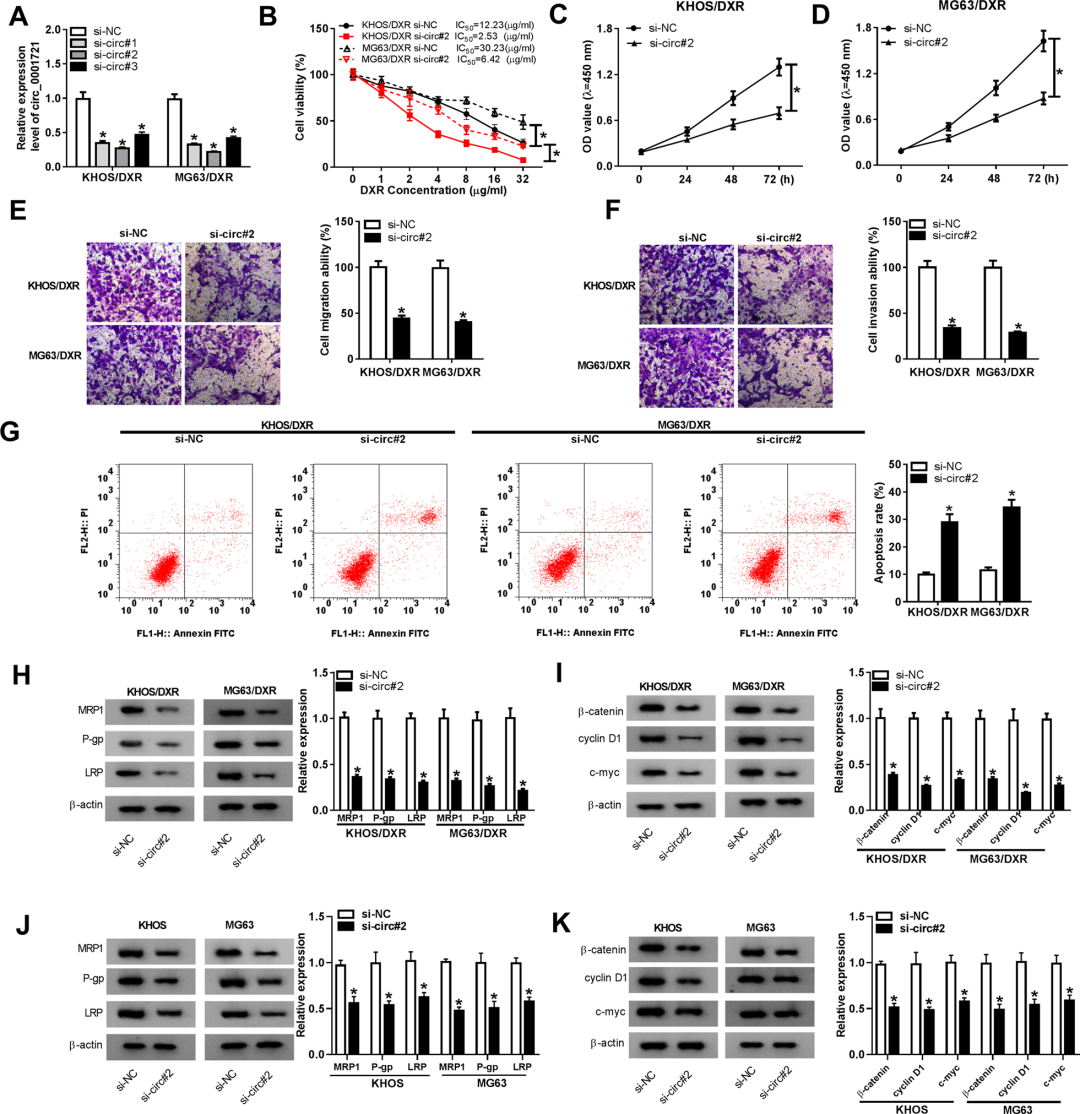
Circ_0001721 knockdown impeded DXR resistance and tumor progression in DXR-resistant OS cells. **A** KHOS/DXR and MG63/DXR cells were introduced with si-NC, si-circ#1, si-circ#2 or si-circ#3, and the knockdown efficiency was examined by qRT-PCR. **B** After DXR treatment with gradient concentration, IC_50_ value was evaluated using CCK-8 assay. **C**, **D** CCK-8 assay was used to assess cell viability. **E**,** F** Transwell assay was utilized to evaluate cell migration and invasion. **G** Flow cytometry was used to monitor the apoptosis rate. **H** The levels of multidrug resistance-related proteins (MRP1, P-gp and LRP) were examined by western blot. **I** The activity of Wnt/β-catenin pathway was evaluated by detecting the protein levels of β-catenin, cyclin D1 and c-myc. **J**, **K** The protein levels of MRP1, P-gp, LRP, β-catenin, cyclin D1 and c-myc were measured in KHOS and MG63 cells transfected with si-NC or si-circ#2. *P < 0.05

To investigate the effect of circ_0001721 on DXR resistance, KHOS/DXR and MG63/DXR cells were introduced with si-NC or si-circ_0001721. Firstly, the transfection efficiency was tested by qRT-PCR, and si-circ#2 with the highest knockdown efficiency was selected for subsequent experiments (Fig. 2A). Then, CCK-8 analysis demonstrated that inhibition of circ_0001721 reduced the IC_50_ of KHOS/DXR and MG63/DXR cells (Fig. 2B). Meanwhile, circ_0001721 silencing resulted in a decrease in the viability of KHOS/DXR and MG63/DXR cells (Fig. 2C, D). Transwell assay showed that transfection with si-circ#2 significantly suppressed cell migration and invasion compared to the si-NC group (Fig. 2E, F). Flow cytometry suggested that knockdown of circ_0001721 overtly enhanced the apoptosis rate of KHOS/DXR and MG63/DXR cells (Fig. 2G). Additionally, we detected the expression of multidrug resistance-related proteins in KHOS/DXR and MG63/DXR cells transfected with si-NC or si-circ#2, and the results indicated that circ_0001721 silence inhibited the protein levels of MRP1, P-gp and LRP (Fig. 2H). Besides, the protein levels of Wnt/β-catenin pathway-related proteins (β-catenin, cyclin D1 and c-myc) were remarkably decreased in KHOS/DXR and MG63/DXR cells introduced with si-circ#2 relative to the si-NC group (Fig. 2I). Meanwhile, circ_0001721 down-regulation significantly reduced the protein levels of MRP1, P-gp, LRP, β-catenin, cyclin D1 and c-myc in KHOS and MG63 cells (Fig. 2J, K). These data unveiled that depletion of circ_0001721 restrained DXR resistance and OS progression via inactivating Wnt/β-catenin pathway.

### Circ_0001721 was a sponge of miR-758

**Fig. 3:**
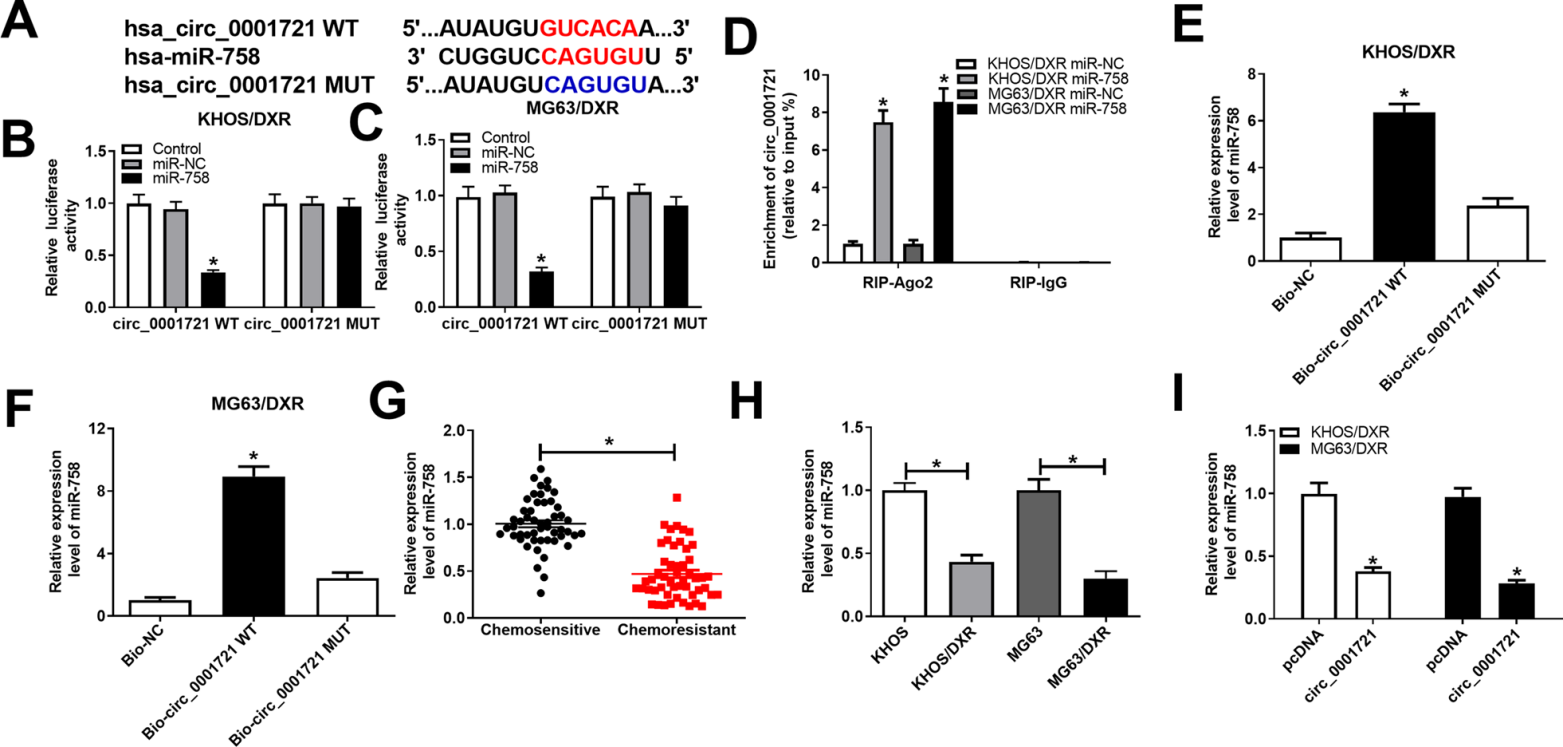
Circ_0001721 was a sponge of miR-758. **A** The putative binding sites of circ_0001721 and miR-758 were exhibited. **B**, **C** Luciferase activity was detected by dual-luciferase reporter assay in KHOS/DXR and MG63/DXR cells co-transfected with circ_0001721 WT or circ_0001721 MUT and miR-758 or miR-NC. **D** RIP assay was performed to confirm whether circ_0001721 bound to miR-758. **E**, **F** RNA pull-down assay was used to verify the relationship between circ_0001721 and miR-758. **G** Expression of miR-758 in Chemosensitive and Chemoresistant OS patients was detected by qRT-PCR. **H** The expression of miR-758 was measured using qRT-PCR in KHOS, KHOS/DXR, MG63 and MG63/DXR cells. **I** The expression level of miR-758 was detected in KHOS/DXR and MG63/DXR cells transfected with pcDNA or circ_0001721. *P < 0.05

We further explored the potential mechanism of circ_0001721 in OS. First, the potential targets of circ_0001721 were predicted through online databases, and the Circular RNA Interactome database showed that circ_0001721 and miR-758 had putative binding sites (Fig. 3A). Next, dual-luciferase reporter assay confirmed that miR-758 mimic strikingly reduced the luciferase activity of circ_0001721 WT reporter (Fig. 3B, C). In addition, RIP and RNA pull-down assays were performed to verify whether circ_0001721 bound to miR-758. RIP analysis showed that circ_0001721 was enriched by Ago2 antibody in KHOS/DXR and MG63/DXR cells transfected with miR-758 (Fig. 3D). RNA pull-down analysis exhibited that miR-758 could be pulled down by Bio-circ_0001721 WT, but not by Bio-circ_0001721 MUT (Fig. 3E, F). Moreover, the expression of miR-758 in the Chemoresistant group was notably lower than that in the Chemosensitive group (Fig. 3G). The level of miR-758 in KHOS/DXR and MG63/DXR cells was observably reduced compared with KHOS and MG63 cells (Fig. 3H). Besides, transfection with circ_0001721 repressed the expression of miR-758 in DXR-resistant OS cells (Fig. 3I). These data indicated that circ_0001721 directly targeted miR-758.

### Inhibition of miR-758 abrogated the effect of circ_0001721 knockdown on DXR resistance in DXR-resistant OS cells

**Fig. 4:**
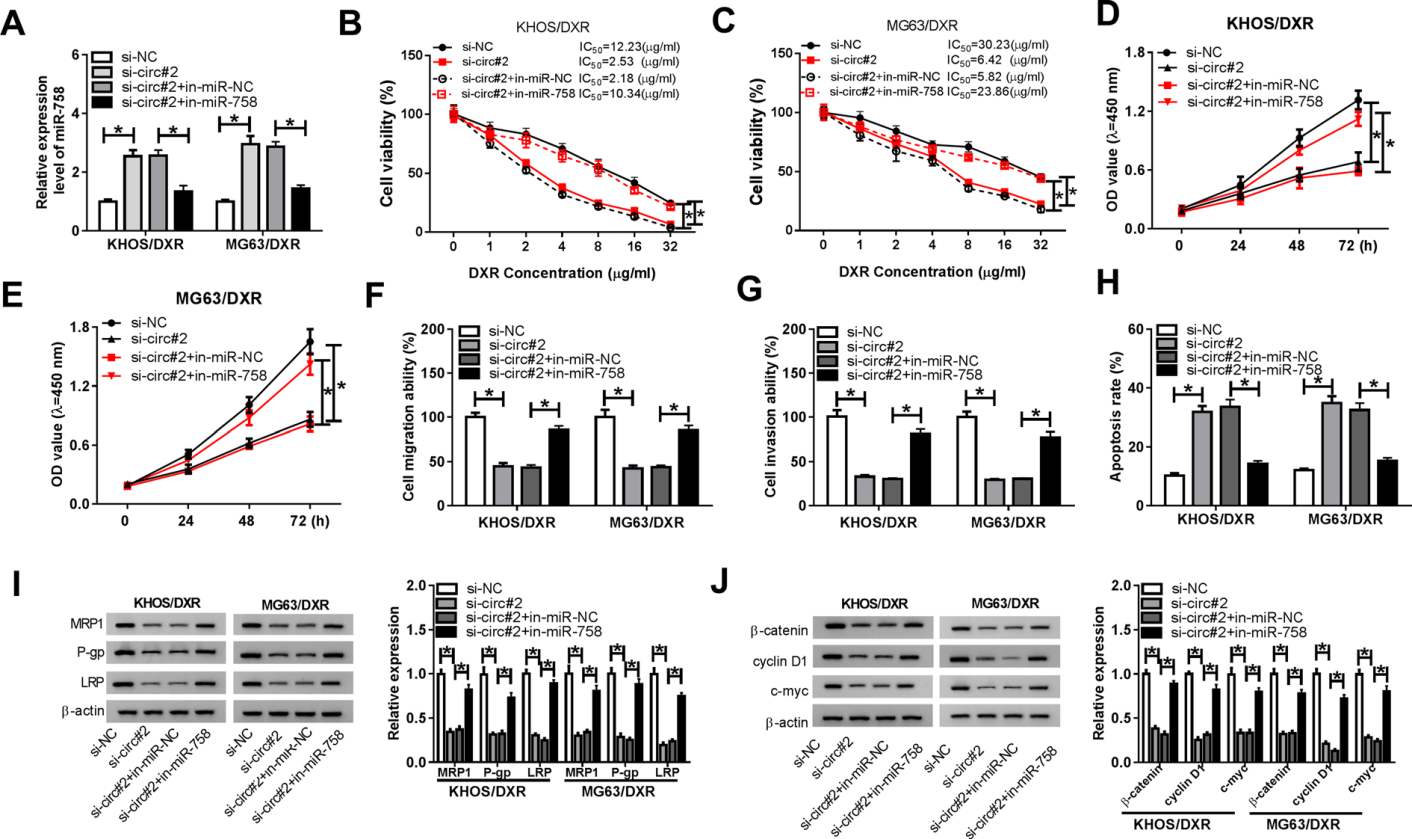
Inhibition of miR-758 abrogated the effect of circ_0001721 knockdown on DXR resistance in DXR-resistant OS cells. KHOS/DXR and MG63/DXR cells were introduced with si-NC, si-circ#2, si-circ#2 + in-miR-NC or si-circ#2 + in-miR-758, respectively. **A** The level of miR-758 was examined by qRT-PCR. **B**, **C** IC_50_ of DXR was determined by CCK-8 assay. **D**, **E** Cell viability was assessed using CCK-8 assay. **F**, **G** Cell migration and invasion were evaluated by transwell assay. **H** The apoptosis rate of KHOS/DXR and MG63/DXR cells was tested by flow cytometry. **I**, **J** The levels of multidrug resistance-related markers and Wnt/β-catenin pathway-associated proteins were examined by western blot. *P < 0.05

Since circ_0001721 was a sponge of miR-758, we speculated that circ_0001721 regulated DXR resistance and OS development by modulating miR-758. In order to verify this assumption, we performed recovery experiments in DXR-resistant OS cells transfected with si-NC, si-circ#2, si-circ#2 + in-miR-NC or si-circ#2 + in-miR-758. First of all, transfection of in-miR-758 reversed the increased expression of miR-758 induced by circ_0001721 silencing (Fig. 4A). CCK-8 analysis suggested that the decrease in DXR resistance caused by circ_0001721 knockdown was abolished by miR-758 down-regulation (Fig. 4B, C). Also, depletion of circ_0001721 decreased cell viability, migration and invasion, while the effects were undermined after introduction with in-miR-758 (Fig. 4D, G). Flow cytometry showed that miR-758 inhibition reversed the effect of circ_0001721 knockdown on cell apoptosis (Fig. 4H). Western blot assay exhibited that circ_0001721 silencing reduced the levels of multidrug resistance-related markers and Wnt/β-catenin pathway-related proteins, whereas miR-758 down-regulation abolished the effects (Fig. 4I and J). These data manifested that circ_0001721 modulated DXR resistance and tumor progression by sponging miR-758 through regulation of Wnt/β-catenin pathway in DXR-resistant OS cells.

### TCF4 was a target of miR-758

**Fig. 5:**
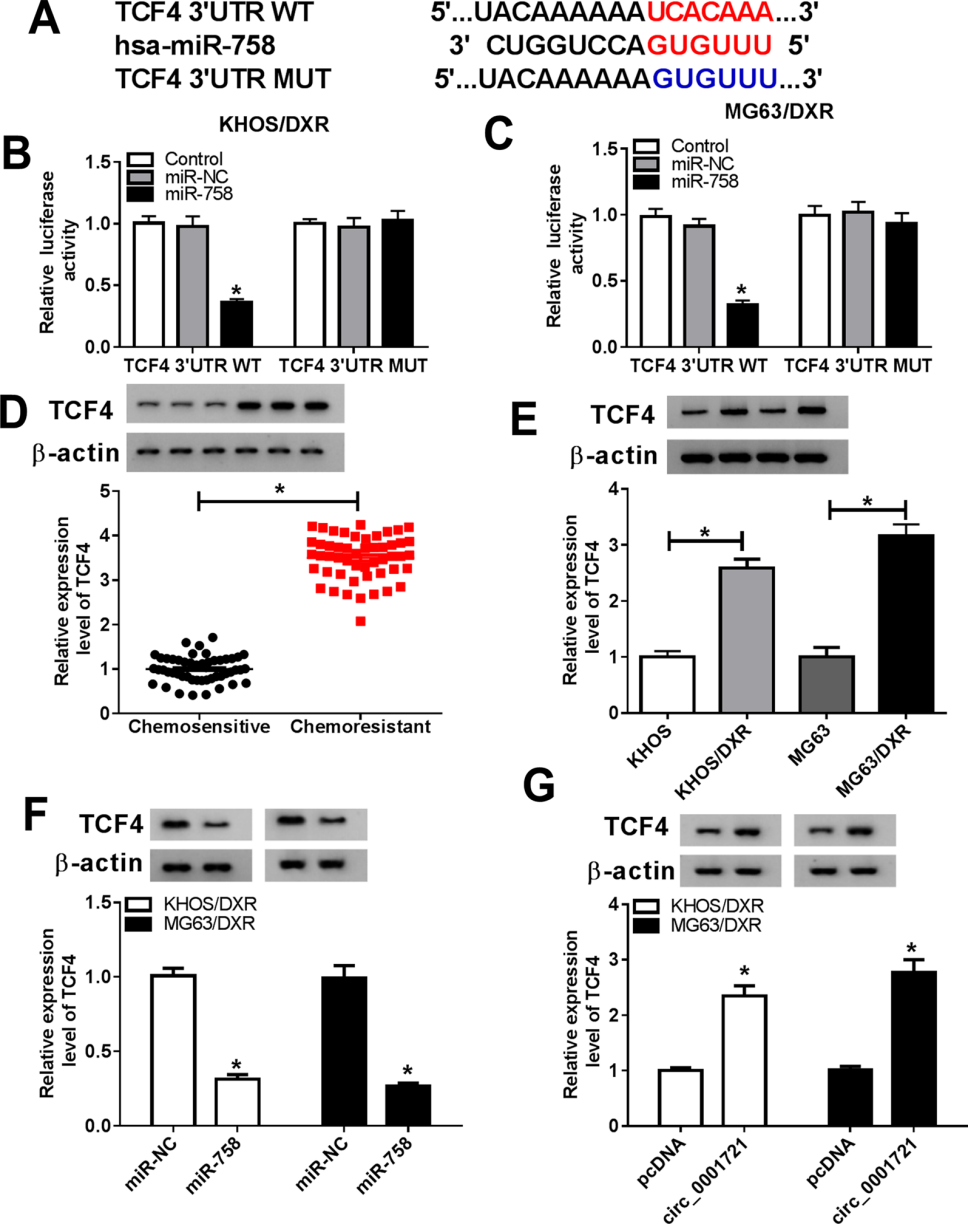
TCF4 was a target of miR-758. **A** The predicted binding sites of miR-758 and TCF4 3’UTR were displayed. **B**, **C** Dual-luciferase reporter assay was performed to validate the relationship between miR-758 and TCF4. **D** The expression of TCF4 was tested in Chemosensitive and Chemoresistant OS patients by western blot. **E** The protein level of TCF4 was detected in KHOS, KHOS/DXR, MG63 and MG63/DXR cells. **F**, **G** KHOS/DXR and MG63/DXR cells were introduced with miR-NC, miR-758, pcDNA or circ_0001721, and TCF4 expression was measured using western blot. *P < 0.05

The online database microT-CDS predicted that miR-758 had complementary binding sites with TCF4 3’UTR (Fig. 5A). Dual-luciferase reporter assay was performed to verify the relationship between miR-758 and TCF4, and the results revealed that mature miR-758 prominently decreased the luciferase activity of TCF4 3’UTR WT reporter (Fig. 5B, C). In addition, the protein level of TCF4 was overtly increased in the Chemoresistant group relative to the Chemosensitive group (Fig. 5D). Similarly, TCF4 was upregulated in KHOS/DXR and MG63/DXR cells relative to KHOS and MG63 cells (Fig. 5E). Besides, western blot analysis exhibited that miR-758 mimics inhibited TCF4 expression, and circ_0001721 overexpression promoted TCF4 expression (Fig. 5F, G). These data evidenced that TCF4 was a target of miR-758.

### TCF4 alleviated the inhibition of miR-758 on DXR resistance in DXR-resistant OS cells

**Fig. 6:**
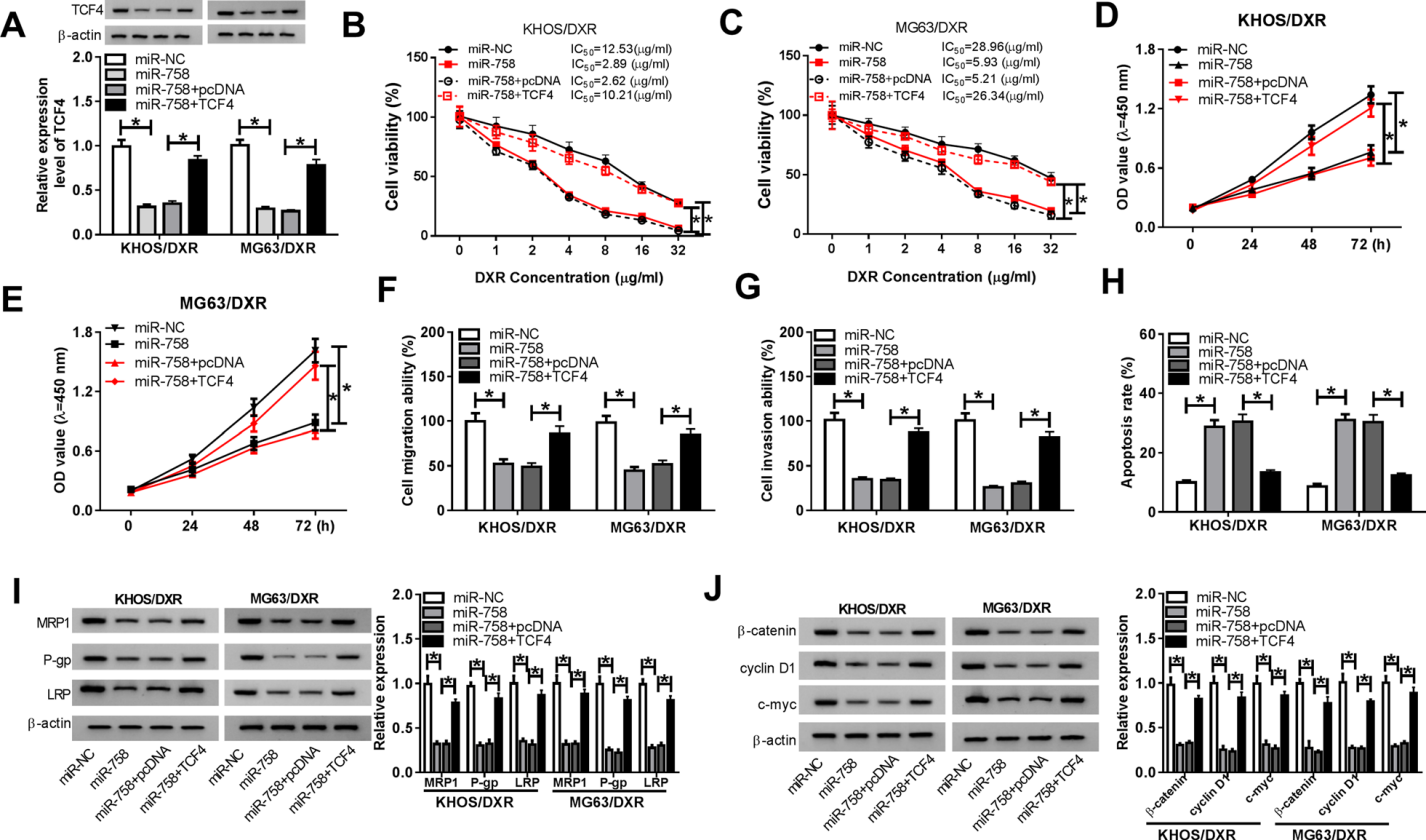
TCF4 alleviated the inhibition of miR-758 on DXR resistance in DXR-resistant OS cells. KHOS/DXR and MG63/DXR cells were transfected with miR-NC, miR-758, miR-758 + pcDNA or miR-758 + TCF4, respectively. **A** The protein expression of TCF4 was detected using western blot. **B**, **C** After treatment with different concentrations of DXR, IC_50_ was determined by CCK-8 assay. **D**, **E** CCK-8 analysis was used to assess cell viability. **F**, **G** Transwell assay was utilized to determine cell migration and invasion. (H) The cell apoptosis rate was monitored by flow cytometry. **I** and **J** The levels of multidrug resistance markers and Wnt/β-catenin pathway-related proteins were detected by western blot. *P < 0.05

To elucidate the correlation between miR-758 and TCF4 in DXR resistance and OS progression, KHOS/DXR and MG63/DXR cells were introduced with miR-NC, miR-758, miR-758 + pcDNA or miR-758 + TCF4. Firstly, transfection of TCF4 rescued the decrease in TCF4 expression caused by miR-758 upregulation (Fig. 6A). Furthermore, upregulation of TCF4 abolished increased DXR sensitivity caused by miR-758 overexpression (Fig. 6B, C). CCK-8 assay and transwell assay revealed that the viability, migration and invasion of KHOS/DXR and MG63/DXR cells were dramatically suppressed by miR-758 mimics, which were reversed by upregulating TCF4 (Fig. 6D, G). Besides, transfection with miR-758 increased the apoptosis rate of KHOS/DXR and MG63/DXR cells, while the effect was abrogated by overexpressing TCF4 (Fig. 6H). Moreover, the protein levels of multidrug resistance-related and Wnt/β-catenin pathway-related proteins were decreased in DXR-resistant OS cells transfected with miR-758 mimics compared with the miR-NC group, whereas the levels were reversed by increasing TCF4 (Fig. 6I, J). These data concluded that miR-758 suppressed DXR resistance and tumor progression via targeting TCF4 through inhibition of Wnt/β-catenin pathway in DXR-resistant OS cells.

### Depletion of circ_0001721 hindered tumor growth in vivo

**Fig. 7:**
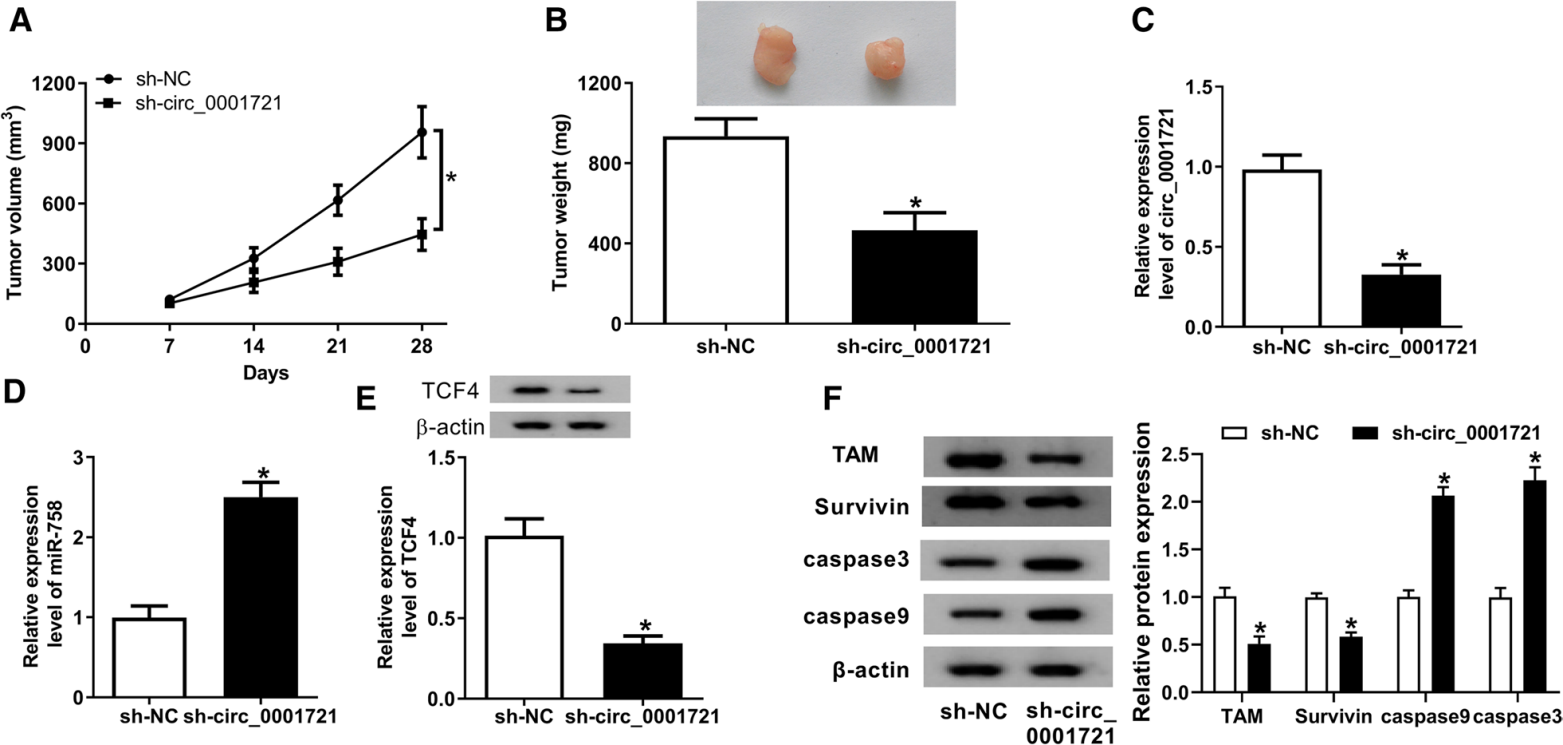
Depletion of circ_0001721 hindered tumor growth in vivo. MG63/DXR cells introduced with sh-NC or sh-circ_0001721 were subcutaneously inoculated into nude mice. **A** After injection, tumor volume was measured once a week. **B** Mice were sacrificed after 28 days, and the xenografts were weighed. **C**–**E** The levels of circ_0001721, miR-758 and TCF4 were detected by qRT-PCR or western blot. **F** The protein levels of TAM, Survivin, caspase3 and caspase9 were examined using western blot analysis. *P < 0.05

We also established a xenograft model to explore the effect of circ_0001721 on the growth of OS in vivo. As displayed in Fig. 7A, B, compared with the sh-NC group, the tumor volume and weight of the sh-circ_0001721 group were observably reduced. In addition, the expression of circ_0001721, miR-758 and TCF4 in xenografts was determined using qRT-PCR or western blot. The results exhibited that silencing of circ_0001721 repressed circ_0001721 and TCF4 levels and elevated miR-758 level (Fig. 7C–E). Also, circ_0001721 knockdown decreased the protein level of TAM and Survivin and increased the protein level of caspase3 and caspase9, suggesting that circ_0001721 silencing induced tumor necrosis and apoptosis in vivo (Fig. 7F). These data indicated that circ_0001721 depletion blocked the growth of xenograft tumors.

## Discussion

Differences in efficacy and toxicity of chemotherapy have become major factors limiting the prognosis of osteosarcoma [20]. Besides, the efficiency of DXR in OS is often limited by acquired resistance [21]. Moreover, the emergence of multidrug resistance (MDR) makes cancer treatment trickier, and several MDR-related genes have been identified, such as MRP1, P-gp and LRP [22]. Recently, non-coding RNAs have been playing an increasingly important role in overcoming complex mechanisms of drug resistance [23]. In this research, we first clarified the effect of circ_0001721 on DXR resistance in osteosarcoma.

Emerging evidence suggested that cytoplasmic circRNAs can mediate target mRNA expression by acting as competitive endogenous RNAs (ceRNAs) and miRNA sponges [24]. For example, circ_0001658 contributed to the development of OS by serving as ceRNA for miR-382-5p to activate YB-1 [25]. Furthermore, circPVT1 silencing decreased OS cell resistance to DXR and cisplatin by repressing ABCB1 (P-gp) [26]. Also, Li et al. discovered that circ_0001721 expedited OS progression by serving as ceRNAs of miR-569 and miR-599 [13]. Consistent with previous studies, circ_0001721 expression was drastically increased in OS. Moreover, circ_0001721 was highly expressed in DXR-resistant patients and cells. Functional experiments demonstrated that circ_0001721 depletion reduced DXR resistance and suppressed tumor development in DXR-resistant OS cells.

To investigate the molecular basis of circ_0001721 in OS, we used bioinformatics to predict miRNAs that might bind to circ_0001721 and selected miR-758 as a candidate. A growing number of researches certified that miR-758 was an inhibitor in many tumors. For instance, Song et al. revealed that miR-758 curbed the progression of cervical cancer via inhibiting HMGB3 through regulation of Wnt/β-catenin pathway [27]. Zhou et al. suggested that miR-758 targeted HMGB3 to hinder the malignant phenotype of non-small cell lung cancer [28]. Meng et al. disclosed that miR-758 blocked tumor metastasis in cervical cancer by targeting MEPE [29]. Additionally, miR-758 impaired OS progression via repressing HMGA1 expression through inactivation of Wnt/β-catenin pathway [30]. In the present study, miR-758 was down-regulated in DXR-resistant tissues and cells. Further, inhibition of miR-758 reversed the effect of circ_0001721 silencing on DXR resistance and tumor progression in DXR-resistant OS cells.

The canonical Wnt/β-catenin pathway participates in the mechanism of tumor cell formation and development [31]. When the Wnt pathway is activated in cancer, increased β-catenin causes cyclin D1 and c-myc to be activated [32]. Blocking the small molecules that interact between β-catenin and TCF4 can effectively impede Wnt/β-catenin signal transduction, thereby digging out new anticancer drugs [33]. In the current study, TCF4 was remarkably elevated in DXR-resistant tissues and cells. More importantly, TCF4 reversed the inhibitory effect of miR-758 on DXR resistance in DXR-resistant OS cells.

In conclusion, circ_0001721 increased DXR resistance and promoted tumor progression in OS via regulating miR-758/TCF4 axis through Wnt/β-catenin pathway. These findings suggested a new therapeutic marker for chemotherapy of osteosarcoma. The limitation of this work is the lack of in vivo studies on doxorubicin resistance. In addition, the exact molecular mechanism of circ_0001721 in osteosarcoma needs to be explored in future research.

## Data Availability

Not applicable.
